# Medical Students’ Knowledge and Perception of Anesthesia: An Insight Into Anesthesiology as a Career Choice

**DOI:** 10.7759/cureus.53819

**Published:** 2024-02-08

**Authors:** Fahad N Alrajban, Saad I Aldraihem, Mallek M Alghamdi, Sarah B Delvi, Omar M Alhalabi, Mohammed Delvi

**Affiliations:** 1 Medicine and Surgery, King Saud University, Riyadh, SAU; 2 Medicine and Surgery, Vydehi Institute of Medical Sciences and Research Centre, Bengaluru, IND; 3 Anesthesiology, King Saud University, Riyadh, SAU

**Keywords:** medical students, role of the anesthesiologist, anesthesiology awareness, anesthesiology knowledge, anesthesia

## Abstract

Introduction

Anesthesia is an important specialty in the medical field responsible for caring for patients before, during, and after operations. It involves monitoring vital signs, managing pain levels, and regulating consciousness. There are various subspecialties of anesthesia, including general anesthesia, intensive care medicine, cardiac anesthesia, and pain medicine, among others. This study aims to assess and evaluate the knowledge and perceptions of medical students regarding the role of anesthesiologists and the factors influencing their career choices.

Methods

A descriptive cross-sectional study was conducted with 379 clinical-year medical students from colleges of medicine across Saudi Arabia. An online questionnaire consisting of 26 items was distributed among the medical students. The questionnaire included sociodemographic characteristics, factors influencing the choice of a career in anesthesiology, and perceptions related to the role of anesthesia. Statistical analysis was performed using RStudio [R Core Team (2021), R version 4.3.1, R Foundation for Statistical Computing, Vienna, Austria]. Categorical variables were presented using frequencies and percentages, while numerical variables were expressed using the median and interquartile ranges (IQRs).

Results

Among the 379 clinical-year medical students surveyed, a majority of participants (59.6%) reported undergoing a mandatory rotation in anesthesia during their fifth or sixth (final) year of medical school. It is noteworthy that good knowledge was significantly associated with having a mandatory rotation in anesthesia during the fifth or sixth year, with 96.0% of students who had a mandatory rotation demonstrating good knowledge, compared to 88.2% of those who did not. A smaller proportion (1.3%) opted for an elective anesthesia rotation during this period. Among those who undertook an elective clinical rotation in anesthesia, all respondents (100.0%) reported undertaking the elective rotation for three weeks or more and stated that this rotation inspired them to pursue a career in anesthesia. Regarding the factors influencing the choice of residency programs, a controllable lifestyle, particularly the ability to control work hours, emerged as the most influential factor, with 96.8% of participants considering it as such. This was followed by income (91.6%), the presence of a doctor-patient relationship (72.6%), and the prestige of the specialty (69.7%).

Conclusion

The medical students demonstrated reasonably good knowledge of the anesthesiologist's role, which can be attributed, in part, to the mandatory rotation in anesthesia. In terms of factors influencing career choice, a good lifestyle was found to be the most influential, followed by income, the doctor-patient relationship, and the prestige of the specialty.

## Introduction

Anesthesiology is a specialized field within the medical profession that plays a critical role in the relief of pain and the provision of care for patients undergoing surgical procedures [1]. Its scope encompasses the administration of anesthesia and the careful management of the patient's vital signs throughout the surgical process. Anesthesia can be broadly classified into three primary categories: general anesthesia, regional anesthesia, and monitored anesthesia care (MAC). General anesthesia is employed when the patient requires complete unconsciousness and unawareness, while regional anesthesia targets specific regions of the body while allowing the patient to remain conscious. Monitored anesthesia care involves the continuous monitoring and regulation of vital signs during a procedure. However, it's important to note that these categories are not always distinct and can overlap in certain situations [1,2].

In the realm of medical education, the selection of specialization areas holds significant importance as it ensures the distribution of physicians across various specialties remains balanced [3]. However, despite its vital role, anesthesiology often remains unfamiliar to many medical students, and the responsibilities of anesthesiologists are not widely comprehended. A Canadian study revealed that less than a third of medical students recognized roles such as acute and chronic pain management, resuscitation, and management of the operating room as responsibilities of anesthesiologists [4]. This lack of awareness presents challenges in attracting medical students to pursue careers in anesthesiology, ultimately resulting in a notable shortage of skilled professionals in this field [3,5].

Highlighting the magnitude of this issue, a recent study conducted locally in Saudi Arabia, which included 248 medical students revealed that a mere 14.5% of them expressed interest in pursuing a career in anesthesia. By comparison, 43.5% indicated an interest in internal medicine, 36.7% in surgery, 27.4% in family medicine, and 23.4% in pediatrics [6].

Understanding the factors that significantly influence students' career choices and improving recognition of anesthesiology and the role of anesthesiologists can help address the shortage in this field [3,7]. Therefore, this study aims to assess medical students' knowledge of anesthesia, the role of an anesthesiologist, and their perceptions of anesthesia as a career in Saudi Arabia.

## Materials and methods

Study design

We conducted a descriptive cross-sectional study using an online survey across colleges of medicine in Saudi Arabia, the data collection process occurred from November 2023 to January 2024 to measure the level of knowledge about anesthesia and the anesthesiologist's role among medical students and perceptions toward anesthesia as a career. The institutional review board approval was obtained from the Research Ethics Committee of King Saud University (application number: HE-23-1079). Their confidentiality was ensured, as there was no personal information or identifier collection in the questionnaire.

Study participants

The selection process targeted male and female undergraduate medical students in Saudi Arabia, implementing specific inclusion and exclusion criteria. Inclusion criteria were defined for clinical years medical students, while pre-clinical years students and interns were excluded from the study.

Sample size

The sample size was estimated using the OpenEpi, Version 3, open-source calculator--SSPropor with a confidence level of 95%, a sample size of 379, and a margin of error of 5%. The questionnaire was sent to 10 colleges of medicine in Saudi Arabia and randomly chosen people. This distribution helped us to receive 379 responses.

Data collection

The tool utilized in this study was a self-generated questionnaire after an extensive review of the literature to measure the level of knowledge about anesthesia and the anesthesiologist's role among medical students and perceptions toward anesthesia as a career. Thirty-seven medical students who were excluded from the main trial participated in a pilot study to pretest the questionnaire, which is the data collection tool, to ensure the quality of the data. After making the necessary adjustments in light of the feedback that was received, the final questionnaire was prepared for the primary study. It consisted of 26 questions, divided into three main sections, student sociodemographic characteristics (five items), factors affecting the choice of anesthesiology as a career (nine items), and perception of the field of anesthesiology (12 items).

Statistical analysis

After obtaining the data, it was reviewed and entered into statistical software for analysis using RStudio [R Core Team (2021), R version 4.3.1, R Foundation for Statistical Computing, Vienna, Austria]. Categorical variables were presented using frequencies and percentages, while numerical variables were expressed using median and interquartile ranges (IQRs). Factors associated with good knowledge levels were examined through a univariable logistic regression analysis, with the knowledge level variable (poor vs. good) as the dependent variable. Independent variables included demographic and academic characteristics, as well as the characteristics of anesthesiology rotations. Statistical significance was set at p < 0.05.

## Results

In the current study, we analyzed data from 379 clinical-year medical students. More than half of the participants were female (51.5%), and the largest proportion belonged to the fifth academic year (43.8%). Regarding the region of their university, a substantial percentage of respondents were from Riyadh (46.2%) (Table 1).

**Table 1:** Demographic and academic characteristics

Questions	N (%)
Gender	
Male	184 (48.5%)
Female	195 (51.5%)
Which academic year?	
4th year	98 (25.9%)
5th year	166 (43.8%)
6th (Final) year	115 (30.3%)
What region is your university in?	
Riyadh	175 (46.2%)
Asir	38 (10.0%)
Makkah	86 (22.7%)
Tabuk	18 (4.7%)
Eastern Region	54 (14.2%)
Others	8 (2.1%)
Residential status	
With family	368 (97.1%)
Without family	11 (2.9%)

A majority of the participants (59.6%) reported having a mandatory rotation in anesthesia during their fifth or sixth year of medical school, while a smaller proportion (1.3%) opted for an elective anesthesia rotation during this period. For those who undertook an elective rotation in anesthesia, all respondents (100.0%) reported undertaking the rotation for three weeks or more, and the unanimous sentiment of these participants was that this rotation inspired them to pursue a career in anesthesia. A small percentage of the study's participants (11.6%) are considering anesthesiology as a future career. Regarding the factors influencing the choice of residency programs, a controllable lifestyle, notably the ability to control work hours, emerged as the most influential factor, with (96.8%) of participants considering it as such. This was followed by income (91.6%), the presence of doctor-patient relationships (72.6%), and prestige of the specialty (69.7%) (Table 2).

**Table 2:** Participants’ perceptions and attitudes toward residency programs, including anesthesiology

Questions	N (%)
Are you considering anesthesiology as a future career?	44 (11.6%)
Do you have a required (mandatory) rotation in anesthesia during your 5th or 6th year of medical school?	226 (59.6%)
Did you take an (elective) anesthesia rotation during your 5th or 6th year of medical school?	5 (1.3%)
If you took a clinical rotation in anesthesia, how long was the rotation?	
3 weeks and more	2 (100.0%)
Missing	1
If you took a clinical rotation in anesthesia, please select the best choice	
This rotation inspired me to pursue a career in anesthesia	2 (100.0%)
I decided against a career in anesthesia	0 (0.0%)
Missing	1
The influence of selected characteristics on the choice of residency programs	
Controllable lifestyle (ability to control work hours)	
Influential	367 (96.8%)
Not influential	12 (3.2%)
Presence of doctor-patient relationship	
Influential	275 (72.6%)
Not Influential	104 (27.4%)
Income	
Influential	347 (91.6%)
Not Influential	32 (8.4%)
Prestige of specialty	
Influential	264 (69.7%)
Not Influential	115 (30.3%)

A notable proportion of participants demonstrated accurate knowledge regarding the anesthesiologist's role across various scenarios. When faced with breathing difficulty either in recovery or in the wards, a significant majority (96.0%) correctly acknowledged the anesthesiologist's role in treating the problem. In terms of the tasks performed by an anesthesiologist in the recovery/ward, a substantial portion (85.0%) recognized their multifaceted responsibilities, encompassing tasks such as inserting a tracheal tube, administering oxygen, and performing tracheostomy. Regarding the anesthesiologist's involvement in different medical settings, a considerable percentage acknowledged their role in the emergency room (73.1%), the care of critically ill patients in the ICU (74.7%), and managing non-surgical pain (68.1%). Furthermore, participants demonstrated a comprehensive understanding of the anesthesiologist's tasks in the ICU, with (80.7%) recognizing their involvement in supporting breathing, resuscitation, and administering sedatives, anesthetics, and analgesics (Table 3). 

**Table 3:** Participants’ responses to items related to their knowledge of the anesthetist role ER: emergency room; ICU: intensive care unit; *An asterisk indicates a correct response.

Questions	N (%)
If you face breathing difficulty either in the recovery or in the wards, do you think the anesthetist has a role in treating your problem?	
No	6 (1.6%)
Yes*	364 (96.0%)
I do not know	9 (2.4%)
What are the possible tasks that can be performed by the anesthetist in the recovery/ward?	
Inserting a tube into your trachea to facilitate your ventilation	11 (2.9%)
Administering oxygen through the face mask	28 (7.4%)
Doing tracheostomy	1 (0.3%)
Has all the above roles*	322 (85.0%)
I do not know	17 (4.5%)
Do you think anesthetists have a role in the ER?	
No	83 (21.9%)
Yes*	277 (73.1%)
I do not know	19 (5.0%)
Do you think the anesthetists have a role in the care of critically ill patients in the ICU?	
No	75 (19.8%)
Yes*	283 (74.7%)
I do not know	21 (5.5%)
What is your opinion regarding the tasks that the anesthetist can do in the ICU?	
Supporting the breathing and ventilation of patients	32 (8.4%)
Has a role in the resuscitation of patients	37 (9.8%)
Administering sedative, anesthetic, and anti pain medications	4 (1.1%)
Has all the above roles*	306 (80.7%)
Do anesthetists have a role in managing non-surgical pain?	
No	88 (23.2%)
Yes*	258 (68.1%)
I do not know	33 (8.7%)

**Figure 1:**
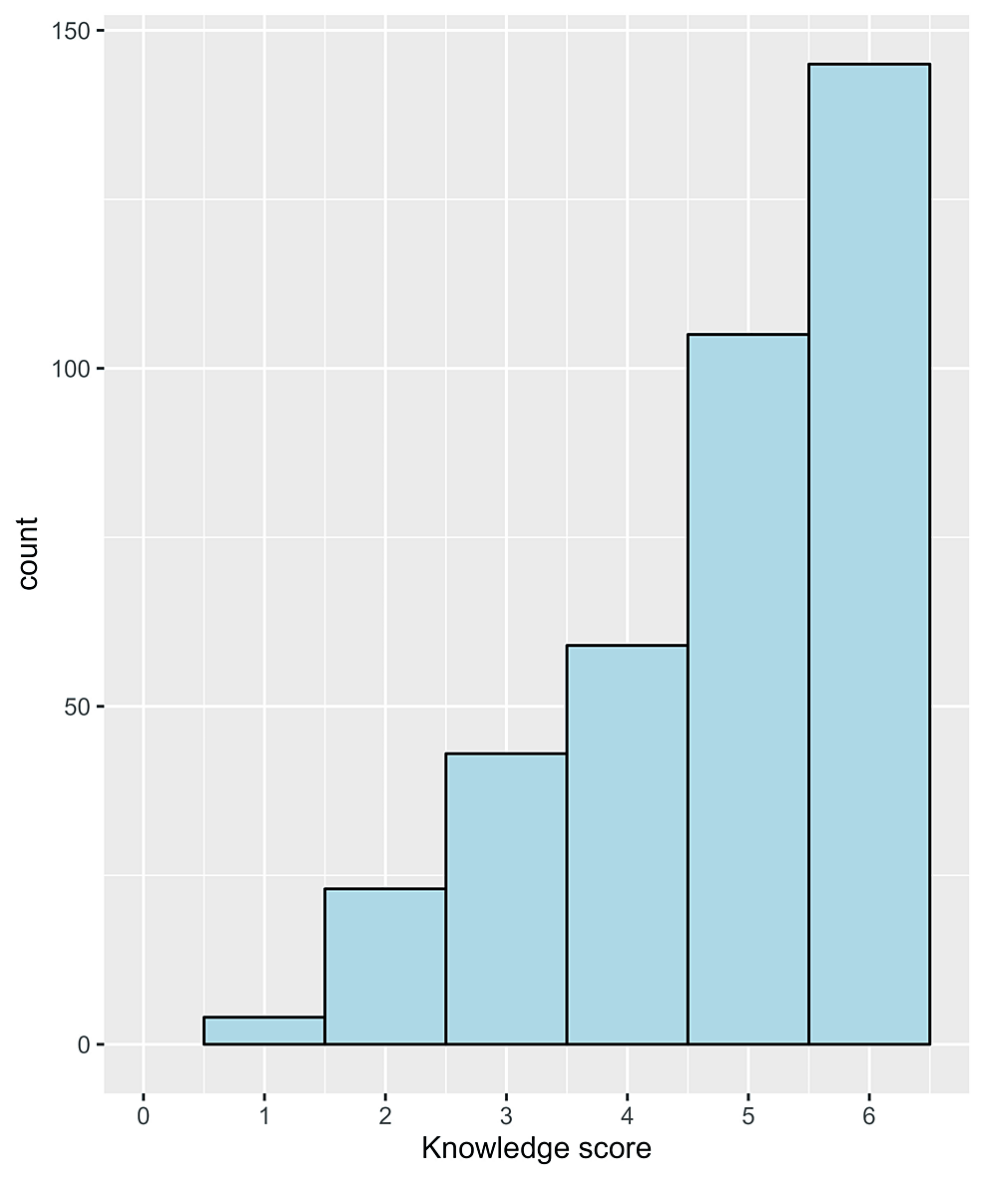
A histogram showing the frequency distribution of the knowledge score

**Figure 2:**
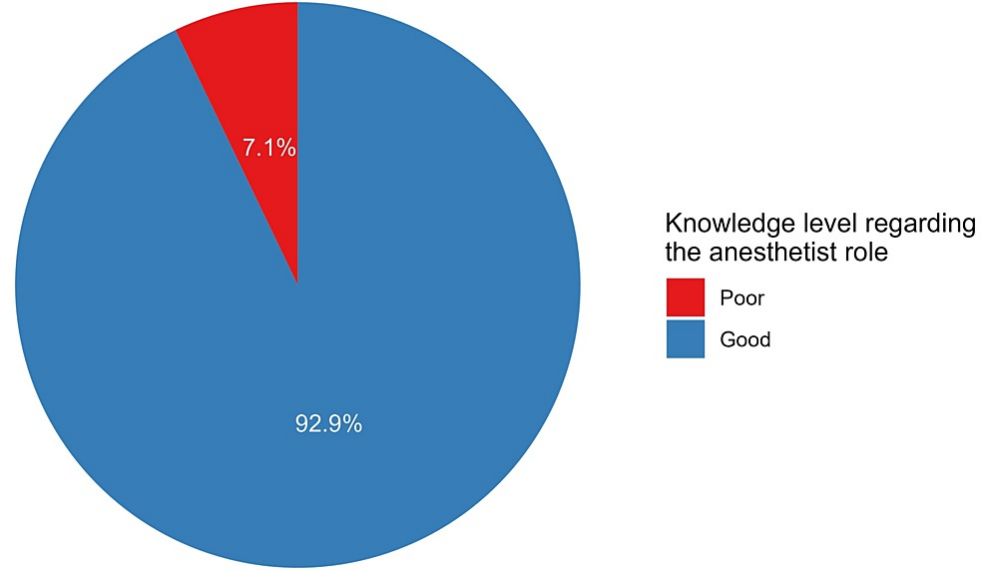
The proportions of knowledge levels about the role of anesthetists

The median raw knowledge score of students was five out of six questions answered correctly (IQR = 4 to 6) (Figure 1). A total of 352 students had adequate knowledge (had ≥50% of correct answers), representing 92.9% of the sample (Figure 2).

Notably, good knowledge was significantly associated with having a mandatory rotation in anesthesia during their fifth or sixth year of medical school, where 96.0% of students who had mandatory rotation had good knowledge compared to 88.2% of those who had not. The odds ratio (OR) of good knowledge was statistically significant [OR = 3.21, 95% confidence interval (CI) 1.44 to 7.69, p = 0.006] (Table 4). Since only one variable was significantly associated with good knowledge, we did not construct a multivariable regression model. 

**Table 4:** Factors associated with good knowledge among students OR: odds ratio; CI: confidence interval; *Data are presented as N (%)

Questions	Knowledge level		Univariable regression		
	Poor N=27*	Good N=352*	OR	95% CI	p-value
Gender					
Male	13 (7.1%)	171 (92.9%)	Reference	Reference	
Female	14 (7.2%)	181 (92.8%)	0.98	0.44, 2.16	0.966
Which academic year?					
4th year	5 (5.1%)	93 (94.9%)	Reference	Reference	
5th year	18 (10.8%)	148 (89.2%)	0.44	0.14, 1.15	0.118
6th (Final) year	4 (3.5%)	111 (96.5%)	1.49	0.38, 6.18	0.559
What region is your university in?					
Riyadh	11 (6.3%)	164 (93.7%)	Reference	Reference	
Asir	6 (15.8%)	32 (84.2%)	0.36	0.13, 1.10	0.058
Makkah	5 (5.8%)	81 (94.2%)	1.09	0.38, 3.54	0.881
Tabuk	0 (0.0%)	18 (100.0%)	NA	NA	0.992
Eastern Region	5 (9.3%)	49 (90.7%)	0.66	0.23, 2.17	0.456
Others	0 (0.0%)	8 (100.0%)	NA	NA	0.995
Residential status					
With family	25 (6.8%)	343 (93.2%)	Reference	Reference	
Without family	2 (18.2%)	9 (81.8%)	0.33	0.08, 2.23	0.168
Are you considering anesthesiology as a future career?					
No	21 (6.3%)	314 (93.7%)	Reference	Reference	
Yes	6 (13.6%)	38 (86.4%)	0.42	0.17, 1.21	0.082
Had a mandatory rotation in anesthesia during your 5th or 6th year of medical school					
No	18 (11.8%)	135 (88.2%)	Reference	Reference	
Yes	9 (4.0%)	217 (96.0%)	3.21	1.44, 7.69	0.006
Took an elective anesthesia rotation during your 5th or 6th year of medical school					
No	27 (7.2%)	347 (92.8%)	Reference	Reference	
Yes	0 (0.0%)	5 (100.0%)	NA	NA	0.990

Students' perceptions toward the role of anesthesiologist revealed interesting insights. The majority of respondents (83.1%) believed that the chief of the operating room was the surgeon, while a smaller percentage (9.8%) identified the anesthesiologist as the chief. Regarding the relationship between the surgeon and the anesthesiologist, a significant proportion (81.0%) perceived that each has different roles. Concerning safety under anesthesia, a substantial majority expressed feeling safe (91.0%). In terms of the perception of an anesthesiologist's role, a considerable number disagreed with the notion that an anesthesiologist is more like a technician than a physician (86.3% disagree or strongly disagree). Furthermore, respondents generally disagreed with the idea that an anesthesiologist is overpaid for their work compared to other medical specialties (74.4% disagree or strongly disagree). On the question of whether an anesthesiologist is overworked compared to other medical specialties, a slight majority (49.3%) were neutral, while a significant proportion agreed or strongly agreed with this statement (37.7%) (Table 5).

**Table 5:** Students’ perceptions towards the role of anesthetists

Questions	N (%)
Who do you think is the chief of the OR?	
The surgeon	315 (83.1%)
The anesthetist	37 (9.8%)
The nurse	6 (1.6%)
I do not know	21 (5.5%)
Description of the relationship between the surgeon and the anesthetist	
Each has different roles	307 (81.0%)
The anesthetist performs his job under the surgeon's order	51 (13.5%)
The surgeon performs his job under the anesthetist's order	10 (2.6%)
I do not know	11 (2.9%)
What do you feel about your safety when you are put under anesthesia?	
I feel safe	345 (91.0%)
I do not have any safety issues	28 (7.4%)
I do not feel any safety	6 (1.6%)
An anesthesiologist's role is more like a technician than a physician	
Strongly disagree	108 (28.5%)
Disagree	219 (57.8%)
Neutral	38 (10.0%)
Agree	12 (3.2%)
Strongly agree	2 (0.5%)
An anesthesiologist is overpaid for the work they do compared to other medical specialties	
Strongly disagree	43 (11.3%)
Disagree	239 (63.1%)
Neutral	81 (21.4%)
Agree	14 (3.7%)
Strongly agree	2 (0.5%)
An anesthesiologist is overworked compared to other medical specialties	
Strongly disagree	4 (1.1%)
Disagree	45 (11.9%)
Neutral	187 (49.3%)
Agree	129 (34.0%)
Strongly agree	14 (3.7%)

## Discussion

The present study aimed to delve into the knowledge and perceptions of anesthesia among a group of 379 clinical-year medical students. By examining their understanding of this crucial specialty and assessing its potential as a career choice, the study sought to provide valuable insights into the field. Our findings paint a generally positive picture, with students demonstrating adequate knowledge regarding the anesthesiologist role and expressing favorable overall perceptions towards the field. The results indicate that the majority of students possessed a good understanding of the anesthesiologist's multifaceted responsibilities, encompassing tasks like airway management, pain management, and critical care support in various settings (including the operating room, recovery room, and the intensive care unit). With (92.9%) the medical students participating in our study having good knowledge of anesthesia as a specialty, this is significantly higher than a previous study in India assessing the awareness of the scope of anesthesiology, in which they found that (68.3%) of the students were aware of the scope of the field [2].

Our findings highlight a significant association between having a mandatory rotation in anesthesia and demonstrating good knowledge, emphasizing the importance of such exposures in solidifying the understanding of the specialty. This aligns with previous studies showing that mandatory clinical rotations in anesthesia effectively enhance students' knowledge of the specialty [8-12]. Even though the students participating in our study demonstrated general good knowledge and understanding of anesthesia, it is important to note that anesthesia remains one of the highly demanded medical specialties not only in Saudi Arabia [13] but also in many other countries worldwide [14-16]. The field of medicine encompasses a multitude of specialties, each with its unique characteristics and opportunities. However, among medical students, certain specialties tend to be more favored and sought-after than others. This observation has been reinforced by a recent comprehensive systematic review study, which examined the preferences of medical students across North America, the European Union, Australia, and New Zealand. The study revealed that surgery and internal medicine stood out as the most desired specialties in these regions [17]. Remarkably, these findings were further substantiated by a study conducted locally [18]. Our study also aligns with the findings of a 2021 study conducted in Saudi Arabia, this local study specifically examined medical students' career aspirations and revealed that a mere (14.5%) of participants expressed interest in pursuing a career in anesthesia [6]. Interestingly, our study indicates a slightly lower percentage, only (11.6%) of medical students are considering anesthesia as a future career.

Despite experiencing a notable surge in popularity and recognition [8], anesthesiology continues to face the challenge of being an unpopular choice among medical students [19,20]. This lack of interest can be attributed, at least in part, to the presence of certain misconceptions identified through our research. Firstly, a discernible minority of the students participating in our study (3.7%) hold the perception that anesthesiology primarily revolves around technical skills, thereby potentially underestimating the intellectual challenges and continuous learning required in the field. This misconception fails to acknowledge the intricate decision-making processes, critical thinking, and problem-solving abilities that anesthesiologists must possess to ensure patient safety and optimal outcomes. The dynamic nature of anesthesiology demands adaptability, quick thinking, and the ability to manage complex medical situations, all of which contribute to the intellectual rigor involved in the specialty [7]. Secondly, there exists a common misperception regarding the leadership dynamics within the operating room team. A substantial portion of students identify the surgeon as the chief authority figure in the operating room, overlooking the pivotal role that anesthesiologists play in patient care and safety during surgical procedures. This misconception reflects a need for a clearer understanding among medical students regarding the collaborative nature of the surgical team, where the anesthesiologist works in tandem with the surgeon and other healthcare professionals to ensure a successful outcome.

The convergence of these misconceptions may deter some highly competitive and spotlight-seeking medical students from considering anesthesiology as a career path. The allure of other specialties that offer more visible and prominent roles within the healthcare hierarchy may overshadow the less publicly visible but equally essential contributions of anesthesiologists [17,18]. These students may have a preference for specialties that afford them more direct patient interaction or a greater sense of individual recognition. In light of these findings, it becomes crucial to address and rectify these misconceptions through targeted educational initiatives and enhanced exposure to the diverse aspects of anesthesiology during medical training. By promoting a comprehensive understanding of the intellectual challenges, leadership responsibilities, and collaborative dynamics within the specialty, we can potentially attract a broader range of medical students and foster a deeper appreciation for the vital role anesthesiologists play in healthcare.

Regarding the factors that influence the choice of a future career, our study identified lifestyle, income, doctor-patient relationship, and prestige, respectively, as being influential when making a career choice. Similarly to a 2018 study conducted locally, lifestyle was the most influential factor in choosing a residency program [3]. These findings, along with interest, income, and job opportunities are also highlighted in multiple studies [17,20].

Our study suggests that medical students generally have a good insight into anesthesiology. Anesthesiology as a specialty encompasses many of the factors that influence choosing a specialty. Gaining insight into the attitudes of medical students regarding their decision-making process when selecting a specialty can serve as a valuable foundation for devising strategies aimed at enhancing the appeal of certain fields experiencing a shortage of healthcare professionals, such as anesthesiology. By doing so, we can effectively address the unequal distribution of medical resources and ensure a more balanced workforce allocation across specialties [17,20].

Limitations

The study sample may not be representative of the broader population or the global context in which the research took place, making it difficult to generalize the findings. The main source of data for this investigation was a self-administered survey provided by the participants, which could be influenced by a desire to conform to social expectations rather than genuine knowledge or beliefs. The research employed a cross-sectional method, which only captured participants' knowledge and attitudes at the time of the survey. To gain a more comprehensive understanding of the development of knowledge and perspectives, longitudinal research would be more suitable.

## Conclusions

The study found that most students had a good understanding of anesthesiologists' responsibilities, including airway management, pain management, and critical care support different clinical settings. Understanding the attitudes and perceptions of medical students toward anesthesia can provide a foundation for developing strategies to increase the attractiveness of specialties experiencing a shortage of manpower, such as anesthesiology. By addressing misconceptions, promoting the intellectual aspects of the specialty, and highlighting the collaborative nature of the surgical team, efforts can be made to enhance the appeal of anesthesiology as a career option. Further research and targeted interventions can help bridge the gap between the demand for anesthesiologists and the number of medical students choosing the field, ensuring a well-balanced distribution of medical resources.
